# Specialized pro-resolving lipid mediators in endodontics: a narrative review

**DOI:** 10.1186/s12903-021-01619-8

**Published:** 2021-05-24

**Authors:** Davy Aubeux, Ove A. Peters, Sepanta Hosseinpour, Solène Tessier, Valérie Geoffroy, Fabienne Pérez, Alexis Gaudin

**Affiliations:** 1 Inserm, UMR 1229, RMeS, Regenerative Medicine and Skeleton, Université de Nantes, ONIRIS, 44042 Nantes, France; 2 Université de Nantes, UFR Odontologie, 44042 Nantes, France; 3 CHU Nantes, PHU4 OTONN44093 Nantes, France; 4 School of Dentistry, The University of Queensland, Brisbane, Australia

**Keywords:** Specialized pro-resolving mediators, Pulpitis, Apical periodontitis, Endodontics, Resolution of inflammation, Therapeutic potential

## Abstract

Endodontics is the branch of dentistry concerned with the morphology, physiology, and pathology of the human dental pulp and periradicular tissues. Human dental pulp is a highly dynamic tissue equipped with a network of resident immunocompetent cells that play major roles in the defense against pathogens and during tissue injury. However, the efficiency of these mechanisms during dental pulp inflammation (pulpitis) varies due to anatomical and physiological restrictions. Uncontrolled, excessive, or unresolved inflammation can lead to pulp tissue necrosis and subsequent bone infections called apical periodontitis. In most cases, pulpitis treatment consists of total pulp removal. Although this strategy has a good success rate, this treatment has some drawbacks (lack of defense mechanisms, loss of healing capacities, incomplete formation of the root in young patients). In a sizeable number of clinical situations, the decision to perform pulp extirpation and endodontic treatment is justifiable by the lack of therapeutic tools that could otherwise limit the immune/inflammatory process. In the past few decades, many studies have demonstrated that the resolution of acute inflammation is necessary to avoid the development of chronic inflammation and to promote repair or regeneration. This active process is orchestrated by Specialized Pro-resolving lipid Mediators (SPMs), including lipoxins, resolvins, protectins and maresins. Interestingly, SPMs do not have direct anti-inflammatory effects by inhibiting or directly blocking this process but can actively reduce neutrophil infiltration into inflamed tissues, enhance efferocytosis and bacterial phagocytosis by monocytes and macrophages and simultaneously inhibit inflammatory cytokine production. Experimental clinical application of SPMs has shown promising result in a wide range of inflammatory diseases, such as renal fibrosis, cerebral ischemia, marginal periodontitis, and cancer; the potential of SPMs in endodontic therapy has recently been explored. In this review, our objective was to analyze the involvement and potential use of SPMs in endodontic therapies with an emphasis on SPM delivery systems to effectively administer SPMs into the dental pulp space.

## Background

The word endodontics comes from the old Greek “endo” meaning “inside” and “odont” for “tooth.” Endodontics is the branch of dentistry concerned with the morphology, physiology and pathology of the human dental pulp and periradicular tissues. Dental pulp is a connective tissue that can trigger an immune response when challenged by different stimuli. In vital teeth, injuries such as dental caries, trauma, operative procedures, and periodontal diseases can lead to pulp inflammation, which is termed pulpitis. Pulpitis represents one of the main emergencies in dental practice, is the most prevalent form of orofacial pain, and has been associated with the prescription and potential overuse of opioid analgesics [1]. Similar to that of many chronic inflammatory diseases, uncontrolled or excessive inflammation during pulpitis can lead to chronic inflammation, scarring, and fibrosis [2]. Teeth are composed of soft connective tissue (dental pulp) enclosed within a mineralized hard tissue envelope (dentin and enamel). This inextensible envelope is responsible for the low compliance of the tooth: tissue swelling associated with pulp inflammation is limited, thereby influencing postinjury events that may ultimately lead to immune events that destroy pulp tissue [3]. Moreover, excessive dental pulp inflammation is a major culprit in the dysregulation of pulp healing and regeneration of injured tissues since the inherent mechanisms are significantly hampered [4].

How to best treat deep carious teeth with inflamed pulp is still controversial [5]. For many years, root canal treatment (RCT), which involves cleaning out all of the pulp from the root canal and filling the root canal system, has been the most appropriate treatment. RCT provides a reliable outcome; however, treated teeth are more brittle than nontreated teeth, especially the molars (hazard ratio, 7:1)[6]. The risk of bacterial dissemination throughout the body (bacteremia) always remains after root canal treatment [7–9]. The available literature on the incidence and diversity of odontogenic infection and the correlation between odontogenic bacteremia and systemic disease justifies the contraindication of such treatments in immunocompromised patients or those at risk for infective endocarditis [10–13].

An alternative approach is to remove only the inflamed portion to maintain and preserve the healing potential of the pulp. The ultimate goal of this concept, so-called vital pulp therapy (VPT) is the preservation of pulp vitality and function. The benefits of VPT include the maintenance of defense mechanisms, pain perception as a warning system, healing and tissue regeneration, completion of root formation in young patients to strengthen thin dentin walls and prevention of long-term complications. Despite these advantages, VPT has not become standard practice among dentists or gained general acceptance in the dental community. VPT outcomes have been difficult to predict due to the variable inflammatory status of the remaining pulp tissue. Indeed, pulp inflammation often remains high, leading to pulp necrosis [5, 14–16]. In most clinical situations, the decision to perform endodontic treatment is justified only by the absence of other available therapeutic tools to limit the immune/inflammatory process that impairs the healing and regenerative potential of the remaining and surrounding healthy tissues.

Thus, it is crucial to identify molecular and cellular agents that can attenuate immune/inflammatory events and promote the rapid return of tissue homeostasis. Blocking the immune response entirely with drugs such as anti-inflammatory or immunosuppressive agents is either ineffective, as the immune response contributes significantly to regeneration or has significant side effects [17]. Extensive work over the past few decades has revealed that the resolution of acute inflammation is critical in avoiding persistent chronic inflammation and supporting repair and regeneration [18]. The resolution of inflammation is coordinated and regulated by a large number of mediators, including SPMs. This review aims to provide fundamental information on the involvement of SPMs in endodontics.

## Main text

### Positive and negative aspects of pulp inflammation

Bacteria are the main and initial cause of inflammation and pulp infection. Carious lesions, trauma, wear and cracks provide bacteria and their byproducts access to the pulp through the dentinal tubules. Odontoblasts are the first cells to contact bacteria and their toxins due to their peripheral location in dental pulp tissue. As other cells (skin and mucosal epithelial cells) play multiple roles, these immunocompetent cells initiate and orchestrate the oral immune response [19]. Odontoblasts are first involved in fighting bacterial invasion and activating innate defense by producing beta-defensins and nitric oxide [20, 21]. In parallel, numerous in vitro studies have also shown that odontoblasts may secrete proinflammatory and immunomodulatory mediators, including chemokines and cytokines: interleukin (IL)-6, IL-10, chemokine C-X-C motif ligand (CXCL)1, CXCL2, CXCL8 (IL-8), CXCL10, and chemokine C–C motif ligand (CCL)2 [22–24].

**Fig. 1:**
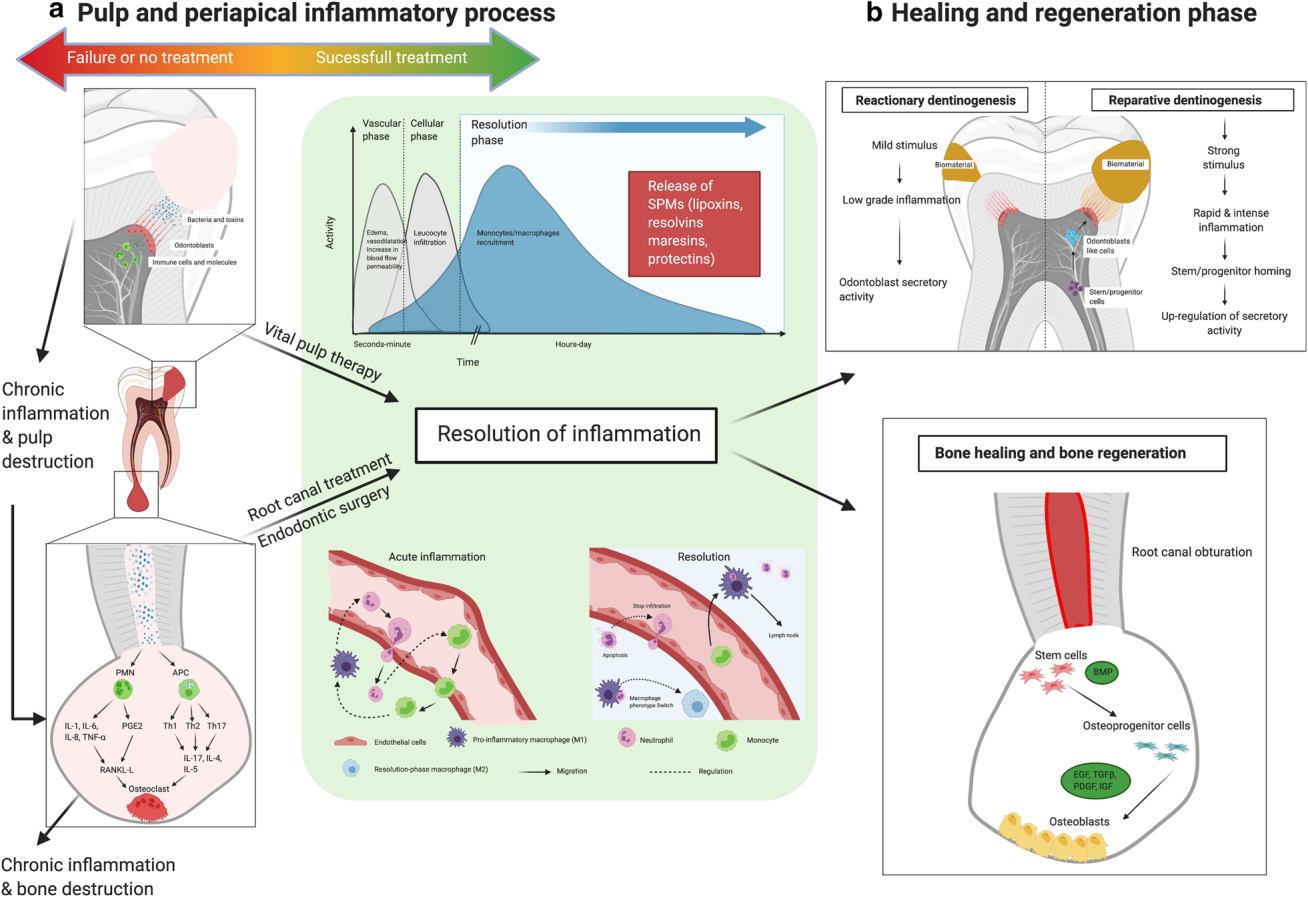
Pulp and periapical inflammatory disease and their ideal outcomes. **a** Pulp and periapical inflammatory process. The pulp inflammatory process is linked to the infiltration of bacteria and toxins. Odontoblasts initiate the immune response. Without therapeutic intervention (upper and lower left panel), inflammation and bacteria cause chronic inflammation, which leads to total pulp necrosis and apical periodontitis. The objective of vital pulp therapies and root canal treatment is to obtain inflammation resolution (central panel). The resolution of inflammation occurs after the first vascular and cellular phases. The resolution of inflammation is coordinated by a large number of mediators termed SPMs. These mediators promote the switch from a proinflammatory (M1) to an anti-inflammatory macrophage phenotype (M2), and nonapoptotic cells ultimately return to the vasculature or lymph. **b** Healing and regeneration phase. Dental pulp has the capacity to produce tertiary dentin when inflammation is resolved. According to the intensity of the stimulus, reactionary or reparative dentinogenesis can be mediated by odontoblasts or odontoblast-like cells, respectively (upper panel). At the apical bone level, the resolution of inflammation allows bone healing and bone regeneration (lower panel)

The low compliance of the pulp environment, the absence of collateral replacement due to terminal vascularization, and the diversity of the microflora at the sites of the carious lesions are responsible for a relative inefficiency of the immune response leading more easily than other tissues to necrosis [25]. Innate immunity plays an important role in superficial caries [26–28] and is responsible for the acute inflammatory response, which is accompanied by systemic vasodilation, vascular leakage, and leukocyte migration. Within a short period after innate immune activation, immune cells (polymorphonuclear neutrophils, odontoblasts and macrophages) secrete various proinflammatory cytokines and chemokines to recruit other immune cells to the site of infection. Neutrophils are the first cells to adhere to endothelial cells and migrate across the vascular wall at the site of infection to engulf invading pathogens and secrete vasoactive and proinflammatory mediators (Fig. 1). In superficial caries, the pulp tissue is not in direct contact with the pathogens; therefore, phagocytosis is not possible. The pulpal responses are subdued and tend to be subclinical. If the innate immune system exceeds its capacity or its defensive effect becomes limited, the adaptive immune system becomes involved, and once activated, specific T and B cells facilitate pathogen clearance. The transition from innate to adaptive immune responses likely occurs in cases of irreversibly inflamed pulp in which the carious lesion front is less than 2 mm from the pulp. In rapidly growing active lesions with pulpal exposure, inflammatory reactions become uncontrolled as the bacteria penetrate the pulp tissue [19, 29].

Until recently, the importance of inflammation in pulpal healing has been underestimated and merely considered an undesirable side effect. New evidence suggests that inflammation is a prerequisite for tissue healing and pulp regeneration. Indeed, the initiated inflammatory response can lead to cell death and tissue destruction or toward wound healing and tissue regeneration. This dynamic balance is linked to the presence and concentration of pro- and anti-inflammatory mediators. A slowly progressing carious lesion can result in pulp healing that is no longer possible if inflammation progresses [29, 30]. Moreover, inflammation can also lead to a cascade of events resulting in excessive immune activity and pulp necrosis. To avoid irreversible damage to pulp tissue, the immune response must be limited to eliminating pathogens without destroying the host tissue (i.e., pulp). The therapeutic aim is to limit tissue damage following injury and control the immune response to promote a return to homeostasis in the dental pulp environment leading to pulp healing.

### Apical periodontitis relies upon a dynamic balance between inflammation and bone resorption

Apical periodontitis (AP) is an inflammatory lesion of the apical and peri-radicular areas mainly caused by bacterial elements from an infected tooth. During pulpitis, bacteria aggregate into a biofilm that adheres to the canal wall. Biofilm development in the root canals is initiated just after the first invasion of the pulp chamber by oral bacteria following inflammatory breakdown of pulp tissue [31]. This biofilm formation starts with surface attachment of bacteria, followed by microcolony development, secretion of extracellular polymeric substances, and then different stages of biofilm maturation and dissociation [32]. Concurrently, the inflammatory lesion front moves toward the apical portion of the root, providing a fluid vehicle for the invading biofilm to progress. The inflammatory response is usually unable to eliminate this biofilm [33]. Consequently, these infections progress to cause total pulp necrosis [34], which stimulates a secondary immune response in the periapical region [35]. As a defense mechanism, AP functions to confine bacteria to the infected tooth and prevent bacterial spread to both adjacent and distant sites. AP is the result of a dual inflammatory reaction that is aggressive and defensive, leading to bone destruction through osteoclastic activation and eventually to root resorption in severe cases [36]. Of note, there is a growing body of evidence showing that several systemic diseases (such as diabetes mellitus or hypertension) may affect the outcome of endodontic treatment and may be considered modulating factors that affect oral infection progression [11, 37–39].

Bacteria exert their pathogenicity through direct and indirect mechanisms. Bacterial factors such as enzymes, exotoxins, peptidoglycan, lipoteichoic acid, and lipopolysaccharides are released into the periradicular tissue, leading to phagocytic influx and the production of proinflammatory mediators. Neutrophils are the first cells involved in the defense and progression of AP, and these cells cause the chemotaxis of monocytes and lymphocytes [36, 40]. Bone macrophages, either resident or recruited from the peripheral blood, secrete inflammatory mediators such as IL-1α, tumor necrosis factor-α (TNF-α), IL-6, IL-8, and transforming growth factor-β (TGF-β) [35] that modulate the inflammatory process. This secretion is implicated in the activation, proliferation, and differentiation of osteoclasts and fibroblasts [41]. Moreover, osteoclasts are also activated by prostaglandins and leukotrienes and, in turn, attract additional polymorphonuclear cells (PMNs) and macrophages to the inflammation site, thus generating an amplification loop [34]. IL-8 and other chemoattractive peptides stimulate leukocyte and monocyte infiltration into periapical lesions. Antigen-presenting cells (APCs), especially dendritic cells (DCs) and macrophages, are crucial in the polarization of T helper (Th) cells toward Th1, Th2, Th17, or T regulatory cells (T regs) [42]. Th17 cells produce IL-17, a proinflammatory cytokine that coordinates with IL-8 to activate the inflammatory process by attracting neutrophils, inducing the production of RANK-L (also known as osteoprotegerin ligand (OPGL)) and activating osteoclasts [40]. RANKL binding to the RANK receptor on the surface of preosteoclasts induces the maturation and activation of these cells, whereas OPG, a naturally occurring inhibitor of RANKL, is a decoy receptor that prevents RANK-RANKL engagement [43, 44].

Advances have been made in understanding the pathogenesis of AP, especially the identification of cells and mediators capable of modulating the immune system. The dynamic balance between the protective effect of inflammation and its destructive capabilities is an active field of investigation that includes immunotherapy [45–47], antioxidants [48–59], selective estrogen receptor modulators [60, 61], stem cells [62–66], phototherapy [67–71], matrix metalloproteinase inhibitors [72, 73], anti-inflammatory agents [74–77], and SPMs [78–81].

### The resolution of inflammation

Historically, the resolution of inflammation has been considered a passive process involving the dilution of chemokine gradients over time; thus, circulating leukocytes no longer sense gradients and are no longer recruited to the site of injury [82]. However, extensive work over the past few decades has revealed that the resolution of acute inflammation is crucial in avoiding the development of persistent chronic inflammation [9]. Resolving inflammation is an active, coordinated, anti-inflammatory, and pro-resolving program that facilitates a return to tissue homeostasis. Although inflammation and its resolution are natural, protective, and active programs inherent in an organism in response to external insult, uncontrolled, excessive, or unresolved inflammation can lead to different forms of tissue damage [83]. In teeth, this inflammation gives rise specifically to scarring and fibrosis in the context of pulpitis and prevents the return to homeostasis [84]. The healing, regeneration, and reconstruction of diseased tissues are significantly hampered [85].

**Fig. 2:**
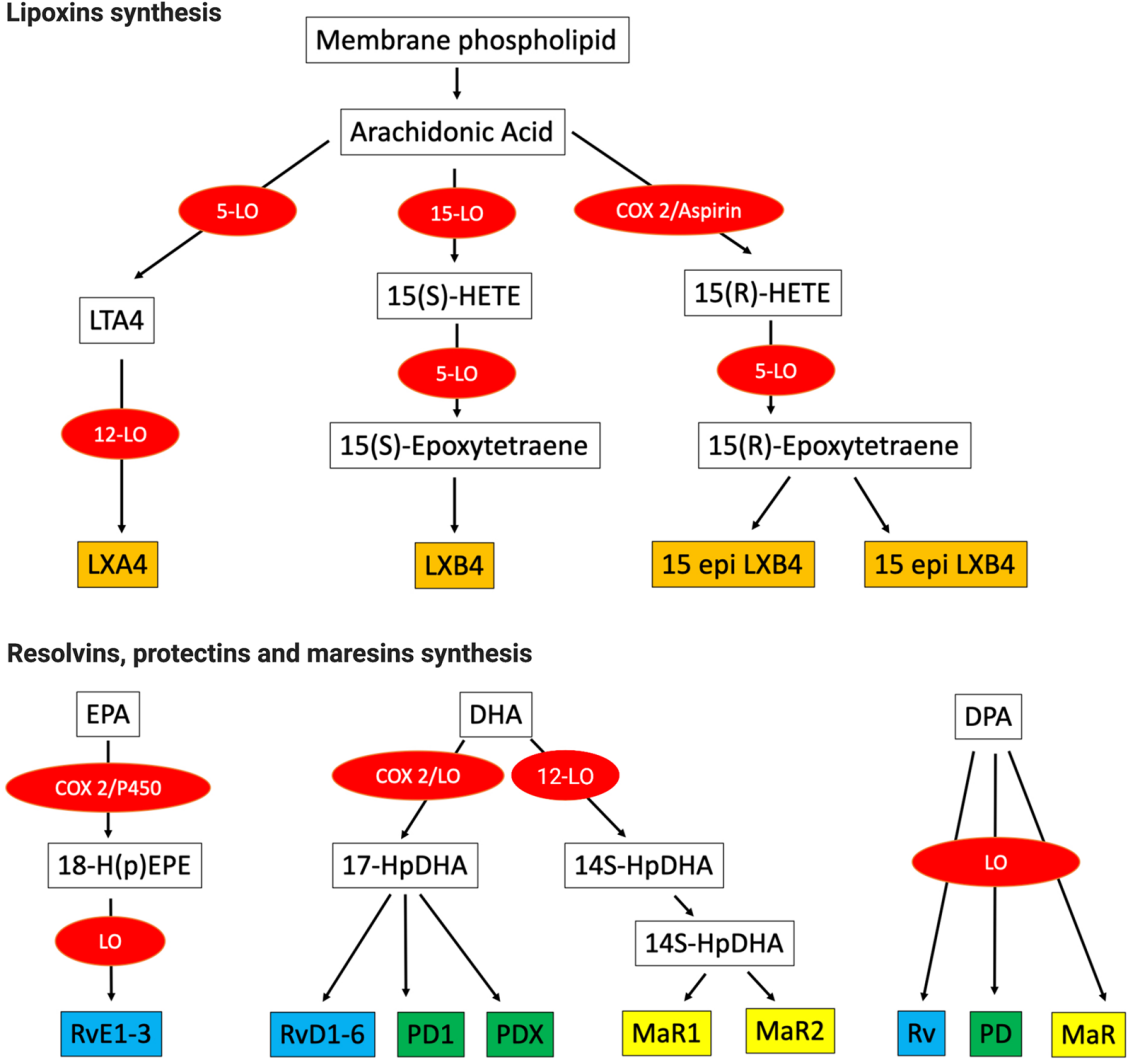
Specialized lipid mediator synthesis. SPMs are all derived from free PUFAs released at the onset of inflammation, including arachidonic acid and ω-3 fatty acids: eicosapentaenoic acid (EPA), docosahexaenoic acid (DHA), and docosapentaenoic acid (DPA). COX, cyclooxygenase; HETE, hydroxyeicosatetraenoic acid; LO, lipoxygenase; LT, leukotriene; LX, lipoxin; H(p)EPE, hydroxyeicosapentaenoic acid; MaR, maresin; PD protectin; Rv, resolvin

The resolution of inflammation is coordinated and regulated by a large number of mediators, including SPMs. ﻿ These mediators are derived from polyunsaturated fatty acids (PUFAs) and bind to G protein-coupled receptors (GPCRs) [86] (Fig. 2). Approximately 20 ligands (e.g., lipoxins, resolvins, maresins, and protectins) and 6 receptors (ALX/FPR2 [87, 88], GPR32, GPR18 [89], chemerin1, BLT1, and GPR37) have been identified to date, highlighting the complex and multifaceted nature of immune resolution [90–92].

### Pro-resolving lipid mediators

#### Lipoxins

Arachidonic acid (AA) synthesis and its derivative products are key regulators involved in the host immune response. The oxygenation of AA initiates the synthesis of powerful bioactive compounds called eicosanoids. These metabolites include prostaglandins, leukotrienes, and lipoxins, which are lipoxygenase (LOX) interaction products. Lipoxin A4 (LXA4) and lipoxin B4 (LXB4) were the first mediators identified that possess specific proresolving actions [93, 94]. These mediators are bioactive autacoid metabolites of omega-6 (ω-6) AA derived by LOX-mediated conversion, followed by the oxidation of 15-hydroxyperoxyeicosatetraenoic acid (15-HpETE).

Lipoxin has three synthetic routes that depend on the cellular environment and result in the formation of lipoxygenase eicosanoid products [95] (Fig. 2). The first route is associated with mucosal and vascular cell–cell interactions. 15-LOX is present in macrophages, monocytes, erythrocytes, and reticulocytes and is induced and activated by IL-4 and IL-13 [96]. Under the action of 15-LOX, an oxygen molecule is inserted on carbon 15 of AA and leads to the formation of ﻿15-HpETE. 15-HpETE serves as a substrate for 5-LOX in neutrophils, which generates an epoxytetraene intermediate that is converted to LXA4 and LXB4. This 5-LOX is regulated by cytokines, including granulocyte–macrophage colony-stimulating factor (GM-CSF) and IL-3 [97].

The second route is linked to platelet-leukocyte interactions. This synthetic route takes place in the vascular system and ﻿involves 12-LOX, which is activated during peripheral blood platelet − leukocyte interactions [98].

Interestingly, the third biosynthesis route is dependent on aspirin and leads to the generation of 15 epi-lipoxin A4, which is also known as aspirin-triggered lipoxin (ATL), and 15 epi-lipoxin B4. Aspirin induces the acetylation of cyclooxygenase-2 (COX-2), transforming AA into 15R-HETE. 15R-HETE is then transformed by leukocytic 5-LOX into 15-epi-LXA4 and 15-epi-LXB4 [99]. The considerable proresolving effects of aspirin compared with those of other nonsteroidal anti-inflammatory drugs (NSAIDs) can be attributed to these processes [100]. Similarly, the clinical benefits and anti-inflammatory effects of statins are associated with the conversion of AA into 15-epi-lipoxin [101].

During inflammatory reactions in vivo, the synthesis of lipid mediators occurs in two stages. In the initial acute phase of inflammation, the leukotriene B4 concentration increases with the infiltration of neutrophils. The accumulation of neutrophils also coincides with increasing prostaglandin E2 (PGE2) concentrations. This prostaglandin induces LOX activity, and the resultant lipoxins contribute to the resolution of inflammation [102, 103].

Lipoxins play multiple roles in the inflammatory response, even at picogram-level concentrations [104]. Lipoxins regulate innate immune system function (e.g., attenuate monocyte recruitment [95]), induce a proresolving M2 phenotype [86, 105], inhibit the functions of DCs [106], and modulate the adaptive immune system by decreasing memory B-cell responses [107]. Lipoxins also inhibit the activation of eosinophils and lymphocytes [108, 109]. In parallel, these factors promote the non-phlogistic infiltration (without the release of proinflammatory mediators) of monocytes and macrophages [100]. Lipoxins stimulate the return of vascular permeability homeostasis and are now recognized as antiedemogenic mediators [110]. Furthermore, lipoxins improve stem cell (periodontal and apical stem cell) proliferation, migration, and wound healing capacity. The binding of LXA4 to its receptor ALX/FPR2 regulates inflammatory mediators (inhibits cytokine, chemokine, and growth factor secretion) and enhances immunomodulatory properties (inhibits the proliferation of T lymphocytes in mixed leucocyte reactions) [88, 111].

#### E- and D-series resolvins

Resolvin (Rv) is an term for resolution-phase interaction products. These factors are natural exudate products defined by their potent bioactivities. These endogenous mediators are synthesized during the resolution phase of inflammation from ω-3 fatty acids, including eicosapentaenoic acid (EPA) to produce E-series resolvins (RvE1, RvE2, and RvE3) and docosahexaenoic acid (DHA) to produce D-series resolvins (RvD1–RvD6), following COX-2 acetylation or cytochrome p450 activity [112] (Fig. 2). Even at pico-nanogram doses, resolvins counteract the proinflammatory state and actively promote resolution via monocyte/macrophage uptake of debris, apoptotic PMNs, and the killing/clearance of microbes [100, 113]. RvE1 reduces dendritic cell IL12 production [114] and potently stimulates IL10 production [115, 116].

RvD1–RvD6 protect against PMN-mediated reperfusion-induced organ injury [117]. RvD2 prevents leukocyte infiltration via the production of nitric oxide and inhibits the production of cytokines, thereby improving the clearance of microbes [118]. RvD5 decreases the amount of bacteria present in circulating blood and exudates, improves phagocytosis, and counteracts proinflammatory markers [100].

#### Maresins and protectins

﻿The biosynthesis of protectins and maresins involves the formation of epoxide intermediates of DHA (Fig. 2) [90]. When produced in neural systems, protectins are termed neuroprotectin D and exert potent protective effects on the retina, brain, and pain [100, 119]. Maresin 1 can reduce proinflammatory cytokines by inhibiting the NF-ĸB pathway and activating M2 macrophages and ﻿is involved in pain mechanisms and tissue regeneration [120].

### Specialized pro-resolving lipid mediators and pain control

There is increasing evidence that SPMs play an important role in the reduction of pain [93, 121]. Some studies, particularly those in rheumatology and gastroenterology, have shown anti-inflammatory effects and resolution leading to the potential control of pain [122, 123]. For example, in an induced rat model of arthritis, it has been shown that resolvin can reduce pain associated with acute or chronic inflammation more effectively than steroid or analgesic treatments [124]. In addition, compared with COX-2 inhibitors, resolvin has been shown to attenuate inflammatory pain via central and peripheral actions in mice [125]. Recent studies have also shown that SPMs can reduce inflammatory pain, postoperative pain and neuropathic pain in animal models via immune, glial and neuronal modulation [126]. SPM receptors (ALX/FPR2, ChemR23) are present on neuronal bodies, nerve terminals and synaptic terminals [127]. Additional studies need to be performed to assess the effect of SPMs on postoperative endodontic pain and to determine whether the results observed in previous studies are translatable to pulpitis or apical periodontitis pain.

### Specialized pro-resolving lipid mediators in endodontics

SPMs have been studied by extensively and their potent effects on reducing inflammation have been documented, along with the development of a wide range of preclinical disease models using validated commercially available SPMs [18]. Most of these studies focused on the role of SPMs in a variety of medical pathologies, such as asthma, Alzheimer’s disease, and rheumatoid arthritis. Furthermore, independent evidence supports the putative role of impaired inflammation resolution mechanisms in periodontal disease [128]. A recent systematic review investigated the biological effects of SPMs on periodontal tissues in animals with experimental periodontitis [129]. ﻿ Six studies from the same research team using an experimental periodontitis model applied RvE1 and lipoxins to treat experimental periodontitis. RvE1 and lipoxins were topically applied to treat experimental periodontitis. The application of SPMs significantly prevented alveolar bone loss and promoted bone regeneration compared with those in the control group. The doses of SPMs and the periods of disease induction varied based on the preclinical model used. Two studies further demonstrated the positive shift in microbial composition (a trend for returning the microbiota to a state associated with health), which was consistent with a positive shift in inflammatory status (﻿a decrease in inflammatory cell infiltration and a reduction in osteoclastic activities), and this effect was regulated by SPMs [129]. Finally, a recent study highlighted the role of maresin 1 and RvE1 in promoting the regenerative properties of periodontal ligament stem cells under inflammatory conditions [130]. However, very few studies (7 PubMed citations) have examined the role of SPMs in pulpal and periapical disease (Fig. 1). One systematic review ﻿investigated the potential of SPMs as an adjunct in the treatment of endodontic infection [131].

#### Role of SPMs in pulp inflammation (pulpitis)

A protective effect of RvE1 was shown in an animal model of pathogen-mediated inflammation (rat dental molar pulp exposed to the oral environment). Histological analysis was performed at 24 and 72 h posttreatment and revealed that RvE1 reduced inflammation and induced milder pulp alterations (a mild cellular inflammatory response and less degenerative alterations of the pulp tissue) than the other treatments. These results also demonstrated the lack of benefit of directly applying corticosteroids to the pulp tissue [80]. Moreover, in another pulpitis model (rat dental incisors exposed to the oral environment), RvE1 was able to attenuate the inflammatory response in the exposed pulp of rat incisors [132]. Interestingly, the expression of ChemR23, a transmembrane receptor to which RvE1 can bind, was upregulated during inflammatory processes and downregulated in inflamed teeth that were treated with RvE1, demonstrating an inhibitory effect against leukocyte infiltration.

#### SPMs in apical periodontitis

Numerous studies support systemic oral administration of omega-3 PUFAs for their beneficial effects on inflammatory diseases, including rheumatoid arthritis, bowel diseases, and chronic periodontitis [133, 134]. A study by El Khouli et al. [135] demonstrated that dietary supplementation with omega-3 significantly reduced inflammatory symptoms such as aphthous stomatitis by altering the cellular functions of leukocytes, blocking proinflammatory cytokine production, and inhibiting the production of AA metabolites, thus dampening the proinflammatory response.

This omega-3 regimen could also be used to treat AP because its pathological mechanisms of action involve comparable inflammatory and cellular mediators. Systemic oral administration of proresolving mediators (omega-3 PUFAs) decreased the expression of the proinflammatory cytokines TNF-α, IL-1β, IL-6, and IL-17 and increased production of the anti-inflammatory cytokine IL-10 in a rat model of AP [78]. These results are consistent with a previous report from the same team [81]. In this earlier study, systemic oral PUFA administration suppressed bone resorption and induced bone regeneration by decreasing osteoclastogenesis and increasing osteoblastogenesis in AP.

In a rat model of apical periodontitis in immature rat teeth, RvE1 promoted root formation and reduced inflammation [136]. Radiographic and histologic analysis at 3 and 6 weeks demonstrated that RvE1 reduced the periapical lesion size compared with that in control teeth (without RvE1 treatment). The periapical inflammatory reaction was reduced in the resolvin group. Finally, radiological root lengths and the thickness of the dentinal walls were also greater in the resolvin group than in the control group. This increase may lead to improved resistance to root fracture. Although these results are promising, the authors emphasize the need for a delivery system that can potentiate the stability of RvE1.

In an animal model of tooth revascularization, RvD2 promoted the resolution of inflammation [79]. RvD2 administration within the root canals of necrotic teeth with apical lesions allowed continued calcification around the root apex and prevented and reversed periapical periodontitis compared with those of the control group without RvD2 treatment. RvD2 reduced overall inflammation by decreasing myeloperoxidase activity in phagocytes and reducing cell infiltration. Moreover, RvD2 treatment increased the expression of the receptor GPR18 (mainly expressed on leukocytes, monocytes, and macrophages) inside and outside root canals [79], underscoring the role of RvD2 in apical closure and tissue regeneration around the apex. In summary, these studies demonstrate that in cases of pulpitis or AP, topical and systemic applications of SPMs stimulate regeneration and resolve inflammation.

### Limits, potential, and therapeutic perspectives

SPMs are highly potent, efficacious endogenous ligands associated with positive outcomes in preclinical animal models. Additional studies are required to assess the safety and efficacy of SPMs as therapeutic agents in humans. The development of stable analogs and drug delivery systems is still in the research stage. SPMs are delicate in their physicochemical nature, require complex chemical synthesis and are prone to metabolic inactivation [90]. Clinical trials with SPM analogs that resist metabolic inactivation are underway but are still in early phases [133]. Topical applications (e.g., nanomicellar solutions) have been advocated to treat ocular inflammation and pain in cataract surgery [137]. Furthermore, an LXA4 agonist exhibited efficacy and safety in a pilot study for children with asthma [138]. Interestingly, the incorporation of SPMs into microparticles or nanoparticles has recently been explored in a few preclinical models of periodontitis and has proven to be an effective approach for decreasing periodontal inflammation and bone loss [139]. However, these particles have some drawbacks, including nonspecific biodistribution, poor water solubility, and limited bioavailability [140].

**Fig. 3:**
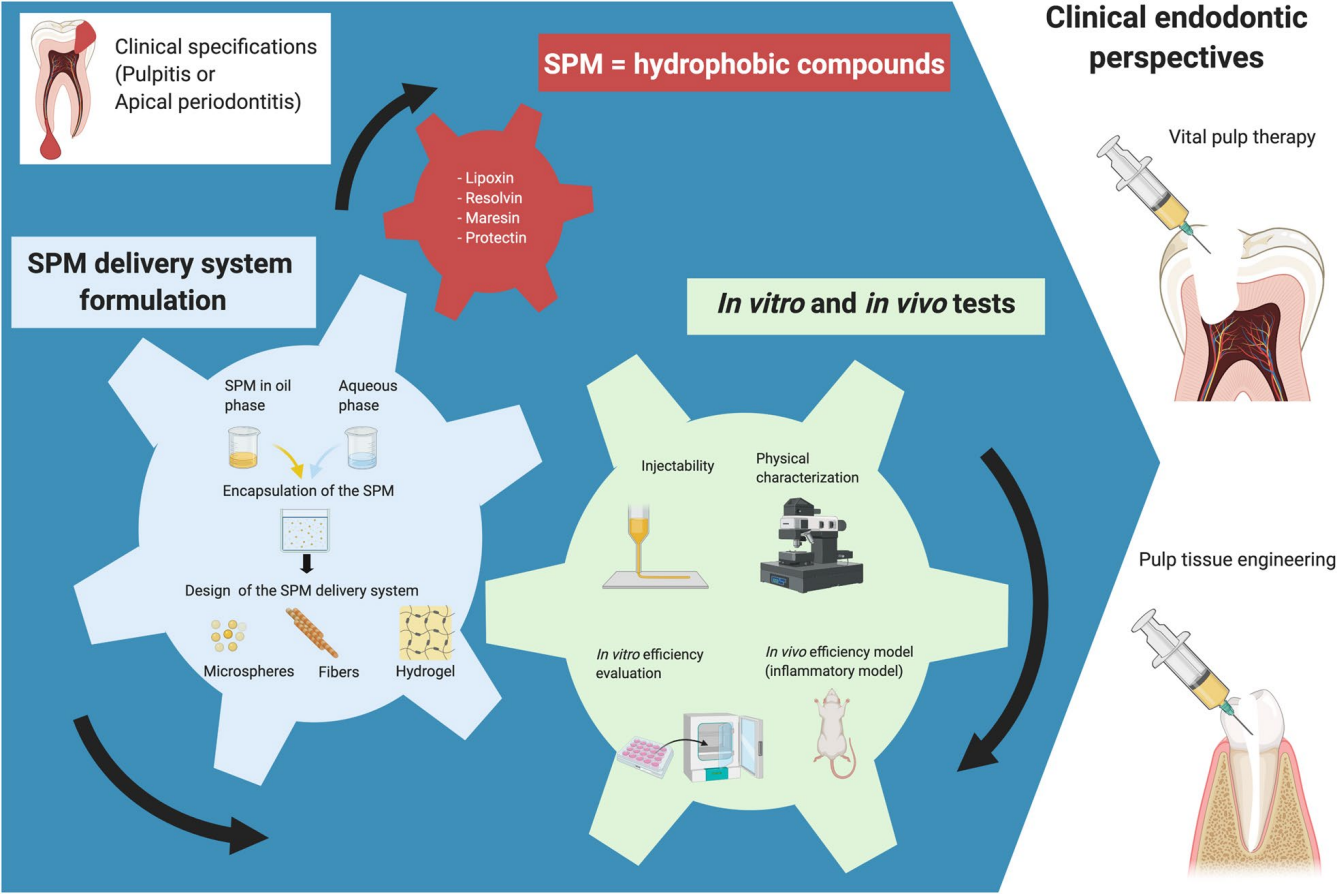
SPM delivery system strategy for endodontic applications. SPMs can be used as therapeutic agents for innovative clinical endodontic therapy for pulpitis or apical periodontitis. To improve SPM efficacy and stability, SPM encapsulation may be required, and different formulations and designs are under investigation (microspheres, fibers or hydrogels) to create new therapeutic devices. Before they can be used in humans, these SPM delivery systems must be characterized and assessed in vitro and in vivo (injectability, physical characterization, efficiency evaluation and animal model assessments) to validate that they fulfill clinical specifications. The clinical objectives are to use these SPM delivery systems for vital pulp therapy or in pulp tissue engineering

To overcome these limitations, different pharmacological approaches are currently under investigation to effectively administer SPMs in the dental pulp space. Access to the dental pulp is generally easier than access to the periapical region. However, the root canal may be narrow with a small volume. Therefore, it is challenging to implant a preformed scaffold into the root canal that would seamlessly cover the entire space of the canal. This concern is one of the main issues that must be addressed when considering drug delivery systems to the dental pulp or the periapical region [141, 142]. The ideal biomaterial should be injectable, biodegradable, biocompatible, and nonimmunogenic, be characterized by high porosity, have adequate mechanical properties, and be easily biofunctionalized or combined with biomolecules such as SPMs. The most common carriers for sustained drug release include microspheres, fibers, and hydrogels [143–145]. Hydrogels are three-dimensional materials that can incorporate a large quantity of water while maintaining their structural and functional integrity. These unique biomedical-grade materials represent a promising delivery system for drugs, proteins, cells, and more because of their biocompatibility, solute permeability and tunable release characteristics (Fig. 3) [146, 147]. Naturally, hydrogels have traditionally been limited to carrying hydrophilic drugs rather than hydrophobic drugs [148]. To improve the drug loading capacity of a hydrophobic compound (e.g., LXA4), different hydrogel formulations are being tested for periodontal applications. ﻿Poloxamer 407 (P407) and ﻿thermoresponsive polyisocyanopeptide (PIC) gels have been compared in vitro; ﻿LXA4 remained bioactive after its release from PIC gels, and no ﻿cytotoxicity was observed for the 1% PIC gel [149] in contrast with other concentrations and P407. In another study, the same team proposed loading LXA4 into acid-terminated, ester-capped ﻿poly(lactic-co-glycolic acid) (PLGA) microspheres using an electrospray procedure combined with PIC gels [150]. In this proof-of-concept study, the authors indicated that ﻿the PIC-PLGA vehicle exhibited suitable injectability, long-term structural stability and no obvious in vivo inflammatory response. Moreover, the ability of SPMs to be incorporated into a scaffold to resolve the inflammatory responses associated with pulpitis and apical periodontitis has yet to be established in the literature.

## Conclusion

Unlike currently available anti-inflammatory drugs, such as anti–TNFα antibodies, SPMs can dampen excessive inflammation without compromising host defense. Furthermore, SPMs do not impair endogenous healing pathways but rather act locally to halt leucocyte recruitment and promote the resolution of the inflammatory response. The few studies that have explored the role of SPMs in endodontics have generated promising results regarding controlling pulp inflammation, promoting the resolution of apical inflammation, and reversing the bone tissue destruction caused by excessive neutrophil activity. Scientific and technical barriers remain due to the instability, complex and delicate physicochemical nature, and potential metabolic inactivation of SPMs. Hydrogel-based drug delivery systems represent a promising approach to overcoming the aforementioned obstacles, and these systems are currently being investigated for periodontal applications in particular. Although the tissues and treatments vary, pulpal and periapical diseases share many features; therefore, advances in SPM delivery systems for periodontal applications will greatly benefit both conditions. This would be an important milestone for modular hydrogel strategies and open a new way to design drug delivery systems for specific applications, such as the treatment of pulpitis and apical periodontitis.

## Data Availability

Not applicable.

## Abbreviations

SPMs: Specialized pro-resolving lipid mediators
RCT: Root canal treatment
VPT: Vital pulp therapy
AP: Apical periodontitis
IL-: Interleukin
CXCL: Chemokine C–X–C motif ligand
CCL: Chemokine C–C motif ligand
TNF-α: Tumor necrosis factor-α
TGF-β: Transforming growth factor-β
PMNs: Polymorphonuclear cells
APCs: Antigen-presenting cells
DCs: Dendritic cells
Th: T helper
T regs: T regulatory cells
RANK-L: Receptor activator of nuclear factor κ B-ligand
OPGL: Osteoprotegerin ligand
PUFAs: Polyunsaturated fatty acids
GPCRs: G protein-coupled receptors
PtdSer: Phosphatidylserine
CRT: Calreticulin
ICAM: Intercellular adhesion molecule
CD: Cluster of differentiation
AA: Arachidonic acid
LOX: Lipoxygenase
LX: Lipoxin
HpETE: Hydroxyperoxyeicosatetraenoic acid
GM-CSF: Granulocyte–macrophage colony-stimulating factor
ATL: Aspirin-triggered lipoxin
COX-2: Cyclooxygenase-2
NSAIDs: Nonsteroidal anti-inflammatory drugs
PGE2: Prostaglandin E2
EPA: Eicosapentaenoic acid
DHA: Docosahexaenoic acid
PIC: Polyisocyanopeptide
PLGA: Ester-capped poly (lactic-co-glycolic acid)
DPA: Docosapentaenoic acid
HETE: Hydroxyeicosatetraenoic acid
LO: Lipoxygenase
LT: Leukotriene
